# The association between empirical dietary inflammatory pattern and risk of cancer and cancer-specific mortality: a systematic review and meta-analysis of prospective cohort studies

**DOI:** 10.3389/fnut.2024.1462931

**Published:** 2024-10-18

**Authors:** Fatemeh S. Hosseini, Ali Nikparast, Elahe Etesami, Fatemeh Javaheri-Tafti, Golaleh Asghari

**Affiliations:** ^1^Department of Nutrition, Science and Research Branch, Islamic Azad University, Tehran, Iran; ^2^Department of Clinical Nutrition & Dietetics, Faculty of Nutrition Science and Food Technology, Shahid Beheshti University of Medical Sciences, Tehran, Iran; ^3^Cancer Research Center, Shahid Beheshti University of Medical Sciences, Tehran, Iran

**Keywords:** EDIP, cancer, cancer-specific mortality, meta-analysis, empirical dietary inflammatory pattern

## Abstract

**Background/aim:**

Current evidence indicates a correlation between the inflammatory potential of diet and the risk of cancer and cancer-specific mortality. This study aimed to assess the association between empirical dietary inflammatory pattern (EDIP), which has recently been designed based on the inflammatory potential of the diet, and the risk of cancer and cancer-specific mortality.

**Methods:**

A systematic literature search was conducted across the PubMed/Medline, Scopus, and Web of Science databases from January 2016 to March 2024. A random effects model was used to calculate the pooled effect size (ES) and 95% confidence intervals (95% CI). Heterogeneity between studies was assessed using the Cochran Q test and the *I*^2^ statistic.

**Results:**

From the initial 229 records, 24 prospective cohort studies with 2,683,350 participants and 37,091 cancer incidence cases, as well as 20,819 cancer-specific mortality, were included in our study. Pooled results indicated a significant association between higher adherence to the EDIP and an increased risk of total cancer (ES: 1.10; 95% CI: 1.05–1.15; *I*^2^ = 41.1), colorectal cancer (ES: 1.19; 95% CI: 1.11–1.27; *I*^2^ = 41.1), and liver cancer (ES: 1.48; 95% CI: 1.14–1.94; *I*^2^ = 36.9). However, no significant association between increased adherence to the EDIP and an increased risk of ovarian or endometrial cancer was found. Furthermore, greater adherence to the EDIP was significantly associated with an increased risk of cancer-specific mortality (ES: 1.18; 95% CI: 1.05–1.33; *I*^2^ = 45.4).

**Conclusion:**

Our results showed that a diet with higher inflammatory properties is associated with an increased risk of cancer and cancer-specific mortality.

**Systematic review registration:**

PROSPERO registration no. CRD42024496912.

## Introduction

The incidence of cancer is increasing at a concerning rate (1), and in 2015, it stood as the second most common cause of death, resulting in over 8.7 million fatalities worldwide (2). Cancer is associated with high rates of disability and premature mortality, also imposing a significant economic burden on healthcare systems (3, 4). Therefore, it is imperative to take a stand and find appropriate approaches to prevent this devastating illness.

According to the body of research, significant evidence supports the notion that low-grade chronic inflammation, characterized by the persistent rise of inflammatory cells and pro-inflammatory mediators, has a considerable impact on the increased risk of cancer and cancer-specific mortality (5). Several inflammatory mediators, such as C-reactive protein (CRP) and cytokines, including IL-1 (interleukin-1), IL-6, and TNF-*α* (tumor necrosis factor-alpha), have been reported to exert carcinogenesis effects through activation of downstream signaling pathways (6). The activation of these pathways may further facilitate angiogenesis and suppress the anti-tumor immune response (5, 6). On the other hand, according to existing research, diet plays an important role in regulating inflammation and the levels of inflammatory cytokines in the bloodstream (7–9). Therefore, assessing the inflammatory potential of diet and its ability to alter and regulate inflammation status can help manage and modify the levels of inflammatory biomarkers. In this context, in the recent decade, the inflammatory potential of diet indicators, such as the dietary inflammatory index (DII) (10), characterized as a literature-derived and population-based indicator, has been evaluated to determine the association between diet-related inflammation and the risk of morbidities such as cancer (11) and mortality (12). This index was developed based on a literature review of original articles between 1950 and 2010 that evaluated the effects of 45 dietary components, including macronutrients, micronutrients, flavonoids, spices, and other bioactive components, on the six most established inflammatory markers, IL-1β, IL-4, IL-6, IL-10 (as an anti-inflammatory component), TNF-*α*, and CRP (13). It should be noted that the DII index has several limitations. The DII is based on nutrients that cannot be accurately estimated due to different food compositions. Moreover, the DII focuses on single nutrients; however, individuals consume nutrients together in the form of food groups.

In the context of this framework, several new indices have been developed, including the Inflammatory Score of the Diet, the Anti-Inflammatory Diet Index, the Dietary and Lifestyle Inflammation Scores, and the Empirical Dietary Inflammatory Pattern (EDIP) (13). Among these, the EDIP, developed by Tabung et al., has attracted increased attention in recent years (14). This diet index is an empirically data-driven dietary pattern score (i.e., derived from a regression model fit a particular dataset) and identifies a dietary pattern according to 18 food groups, which are the most predictive for pro-inflammatory markers, including IL-6, TNF-*α*, and CRP (14). Furthermore, it should be noted that the EDIP has been validated to be highly effective in predicting inflammation biomarkers in three Harvard cohorts (14).

Recently, several observational studies have been conducted to assess the association between adherence to EDIP and the risk of cancer and cancer-specific mortality; however, their results have been inconclusive (15–19). In the Women’s Health Initiative, greater adherence to EDIP was significantly associated with a higher risk of total and site-specific cancer (including colorectal, endometrial, and breast cancer) (15). In contrast, findings from two prospective studies, including the Nurses’ Health Study I and Nurses’ Health Study II, revealed no significant association between greater adherence to EDIP and the risk of endometrial cancer (16). Regarding cancer-specific mortality, Li et al. (17) reported a significant association between greater adherence to EDIP and the risk of cancer-specific mortality; however, other studies did not find any significant association (18, 19). Therefore, our study aimed to conduct a systematic review and meta-analysis of prospective studies to investigate whether adherence to the EDIP can be associated with an increased risk of cancer and cancer-specific mortality.

## Method

### Research registry and standard guidelines

The current study was officially registered on the PROSPERO database with registration ID CRD42024496912 and was conducted in compliance with the PRISMA (Preferred Reporting Items for Systematic Reviews and Meta-Analysis) (see Supplementary Appendix S1) (20) and MOOSE (Meta-analysis of Observational Studies in Epidemiology) guidelines (21). The employed methodology for the search strategy, inclusion and exclusion criteria, and data extraction and analysis procedure were also based on the PECO framework, which stands for Population, Exposure, Comparator, and Outcome (**Table** **1**). Briefly, prospective studies evaluating the association between adherence to the EDIP and the increased risk of total or site-specific cancer (as the primary outcome) and cancer-specific mortality (as the secondary outcome) among adults were included.

**Table 1:** Description of population, exposure, comparator, and outcome (PECO).

Population	Adults (≥18 years old)
Exposure	Highest adherence to EDIP
Comparator	Lowest adherence to EDIP
Outcome	Total cancer or site-specific cancer (primary outcome) Cancer-specific mortality (secondary outcome)

### Data sources and search method

One researcher (EE) employed electronic searches of Scopus, PubMed/MEDLINE, and ISI Web of Science (WOS) databases to perform a comprehensive systematic review of the existing literature. An electronic systematic search was conducted from January 2016 to March 2024 and limited to English publications. We also imposed a time restriction due to the fact that the EDIP score was developed by Tabung et al. and published in 2016 (14). The methods employed for conducting electronic systematic database searches are delineated in Supplementary Table S1. In addition, the same researcher (EE) conducted a thorough manual search of the reference lists of all the included studies and relevant review articles to ensure that our search was comprehensive and that we did not miss any potentially relevant articles.

### Inclusion and exclusion criteria

Studies with the following conditions were included in our systematic review and meta-analysis: (1) individuals who were 18 years of age and above; (2) the study design was prospective; (3) adherence to the EDIP was assessed as the exposure of interest; (4) the outcomes of interest included the risk of total cancer, site-specific, and cancer-specific mortality; and (5) studies reported the adjusted estimates (including odds ratio (OR), hazard ratio (HR), or relative risk (RR)), along with the corresponding 95% confidence interval (CI) as the effect size (ES) for the association between EDIP adherence and the risk of total and site-specific and cancer-specific mortality.

Publications were excluded from our study if there were (1) duplicated studies; (2) systematic review and meta-analysis; (3) clinical trial; (4) case reports, case series, editorials, commentaries, notes, letters, and conference abstracts; (5) animal, *in vivo*, and *in vitro* studies; (6) conducted among newborns, children, adolescents, pregnant mothers, and breastfeeding women population; (7) not available in full-text format; (8) studies that examined our outcome of interest in participants with the disease at baseline; and (9) insufficient data (without relevant ES and 95% CI for our primary and secondary outcomes).

Two investigators (FSH and EE) independently implemented a two-step selection process to find eligible studies. The first stage involved screening titles and abstracts of the identified studies. The second stage involved evaluating the full-length publications deemed relevant. Any discrepancies encountered during the investigation were resolved through constructive discussions with an additional investigator (GA).

### Quality assessment

The quality of the included studies was assessed using the Newcastle-Ottawa Scale (NOS) in the present systematic review and meta-analysis (22). The NOS is a tool that was developed specifically for assessing the quality of non-randomized studies. The assessment of the quality of included studies in this scale is determined by a star system, which is determined by three criteria as follows: (1) selection of the study participants; (2) comparability of the groups; and (3) assessment of either the exposure or outcome of interest. Each item is subject to a maximum rating of one star, except for the comparability item, which can be awarded up to two stars. Publications scoring seven or higher were designated as high quality and low risk of bias. At the same time, those scoring below seven were classified as low quality and high risk of bias publications.

### Data extraction

Two researchers, FJ and FSH, independently conducted an extensive evaluation of each eligible study. The data extracted from eligible studies were as follows: author’s name, publication year, identification of cohort (country and study name), sample characteristics (total and sex-stratified sample size) and number of cases, baseline age and body mass index (BMI) of participants, follow-up duration, person-years, dietary intake assessment method, outcome assessment method, outcome (total cancer, site-specific or cancer-specific mortality), variables adjusted in the multivariate analysis, fully adjusted ES with corresponding 95% CI for risk of total cancer, site-specific or cancer-specific mortality across adherence to EDIP categories. Additionally, any inconsistencies were addressed by discussing with a senior author supervising the work (GA).

### Statistical analysis

Descriptive analysis was used to summarize the characteristics and the demographic details of the participants of the included studies. In order to begin the pooled ES estimate for the risk of total cancer, cancer-specific, or cancer mortality, we used the RR and corresponding 95% CI as the effect sizes for the main analysis. In addition, the HR reported in the original studies was considered equivalent to the relative risks (23). Furthermore, studies that reported effect sizes as odds ratios were converted to relative risk using the formula: RR = OR/[(1 − P0)  + (P0 × OR)], in which P0 indicates the incidence of the outcome of interest in the non-exposed group according to the method of Zhang et al. (24). The pooled ES of total cancer, cancer-specific, or cancer mortality risk were calculated for the highest compared to the lowest adherence as well as per-SD increases in adherence to the EDIP using the DerSimonian and Laird random-effects model, which accounts for variation within and between studies (heterogeneity) (25). The Cochran *Q* test (*P*-heterogeneity) (26) and the *I*^2^ statistic (27) were used to evaluate the proportion of total variation attributable to heterogeneity between studies. When there was variation within the group, the level of significance for Cochran’s *Q* was deemed to be *p* < 0.10 (26). As per the *I*^2^ metrics, 25, 50, and 75% heterogeneity correspond to low, medium, and high degrees of heterogeneity, respectively (28). Subgroup and meta-regression analyses were performed to identify the potential source of the heterogeneity. Subgroup analyses were conducted based on the following factors: age (<55/>55 years), gender (men/women), number of population (<100,000/>100,000), outcome assessment method (medical record/pathologic report), body mass index (normal weight/overweight or obese), baseline type 2 diabetes mellitus (T2DM) status (yes/no), and adjustment for major confounders (total energy intake, supplement used, alcohol consumption, smoking status, physical activity, and family history of cancer). Sensitivity analysis was conducted to evaluate the robustness of the findings and determine whether the final pooled effect sizes were impacted by single or multiple publications using the one-study exclusion (leave-one-out) method. Publication bias was tested by visually inspecting the funnel plot and employing Egger’s regression test (29). All statistical analyses were performed utilizing STATA 17.0 (Stata Corporation, College Station, Texas, USA) software. A *p*-value <0.05 was considered as statistically significant.

## Results

### Literature search

**Figure 1:**
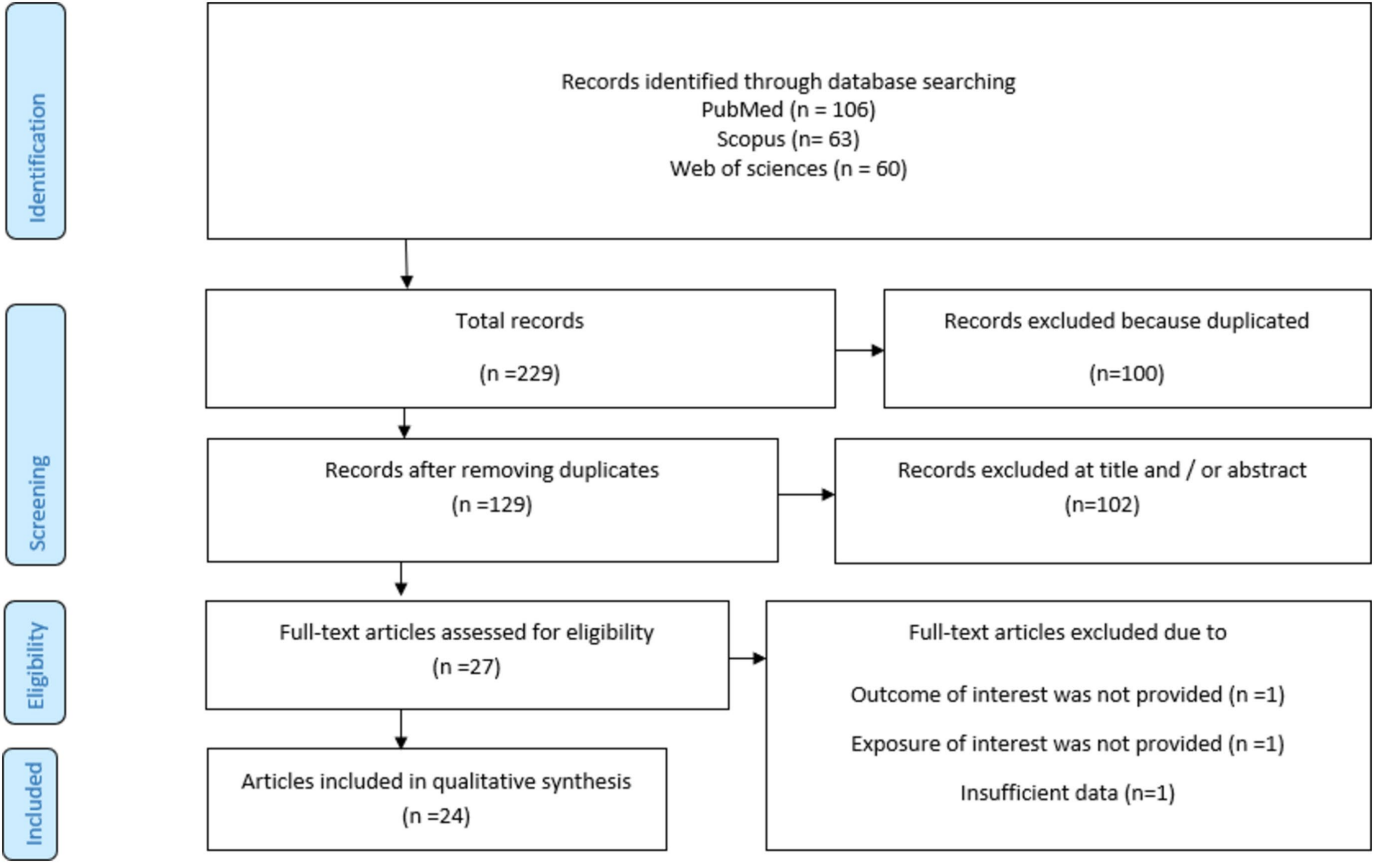
Flowchart of the included studies.

Figure 1 briefly outlines the process of selecting relevant studies and obtaining references from electronic databases. During the initial electronic database search, we identified 229 relevant studies. Of these, 106 were from PubMed/MEDLINE, 63 from Scopus, and 60 from ISI Web of Sciences. After excluding duplicate publications (*N* = 100) and conducting a thorough screening of titles and abstracts to ensure relevance, a total of 27 publications were identified as potentially relevant and underwent comprehensive full-text evaluation. After the full-text evaluation, one study was excluded due to the lack of exposure to our interest, one was excluded due to the lack of outcome, and one was excluded due to insufficient data. Finally, 24 studies were included in our analysis (15–19, 30–48). Out of the 24 included studies, 16 reported the ES for the risk of cancer (15, 16, 31–36, 38–41, 45–48), six reported the ES for cancer-specific mortality, and two reported the ES for both the risk of cancer and cancer-specific mortality (19, 30).

### Characteristics of the included studies

The characteristics of the included studies are presented in Table 2. All the included studies were conducted in the United States and were published between 2017 and 2023, involving a total of 2,683,350 participants. The majority of the included studies were high quality.

**Table 2:** Characteristics of included studies.

First author (year)	Study name (country)	Study duration (follow-up duration, years)	Age, years	BMI, kg/m^2^	Population (men/women)	Number of cases	Outcome	Cancer type	Outcome ascertainment	Dietary intake method	Covariates in fully adjusted model	Quality score
Liu et al. (2017)	HPFS, NHS (United States)	HPFS (26 max), NHS (28 max)	52.21 (mean)	25.25 (mean)	124,433 (47,416/77,017)	1,311	Cancer risk	Colorectal cancer	Medical records	FFQ	Age, family history of colorectal cancer, smoking status, total alcohol intake, PA, BMI, total energy intake, supplement use, aspirin use, endoscopy status.	9
Tabung et al. (2017)	NHS, NHS-II (United States)	NHS (28 max) NHS-II (22 max)	54.38 (mean)	26.54 (mean)	186,314 (0/186,314)	989	Cancer risk	Ovarian cancer	Medical records	FFQ	Age, calendar time, parity, family history of breast cancer or ovarian cancer, BMI, total energy intake, supplement use, duration of breastfeeding, duration of oral contraceptive use, menopausal status, postmenopausal hormone duration, tubal ligation, hysterectomy.	9
Liu et al. (2018)	HPFS, NHS (United States)	HPFS (26 max), NHS (28 max)	52.19 (mean)	25.22 (mean)	124,433 (47,416/77,017)	951	Cancer risk	Colorectal cancer	Pathophysiologically confirmation	FFQ	Family history of colorectal cancer, smoking status, total alcohol intake, PA, BMI, total energy intake, supplement use, aspirin use, and endoscopy status.	8
Tabung et al. (2018)	HPFS, NHS (United States)	HPFS (24 max), NHS (26 max)	62.61 (mean)	25.7 (mean)	121,050 (46,894/74,246)	2,699	Cancer risk	Colorectal cancer	Medical records	FFQ	Age, race, family history of cancer, smoking status, total alcohol intake, PA, total energy intake, supplement use, aspirin use, NSAID use, endoscopy status, menopausal status, and postmenopausal hormone use.	6
Lee et al. (2019)	HPFS, NHS (United States)	HPFS (28 max), NHS (30 max)	52.19 (mean)	25.24 (mean)	116,983 (47,232/69,751)	478	Cancer risk	Multiple myeloma	Medical records	FFQ	Age, BMI, and total energy intake	7
Abufaraj et al. (2019)	HPFS, NHS, NHS-II (United States)	HPFS (26 max), NHS (28 max), NHS-II (22 max)			342,264 (218,074/12,4190)	1,042	Cancer risk	Bladder cancer	Medical records	FFQ	Age, smoking status, total energy intake, total fluid intake, aspirin use, NSAID use, menopausal status, age at menopause, and pregnancy.	7
Aroke et al. (2020)	PLCO (United States)	12.14 (median)	62.5 (mean)	27.5 (mean)	49,317 (49,317/0)	4,176	Cancer risk	Prostate cancer	Pathophysiologically confirmation	FFQ	Age, race, education, occupation, family history of cancer, smoking status, PA, BMI, total energy intake, aspirin use, ibuprofen use, use of PSA screening tests, PLCO study center, chronic disease comorbidity score.	7
Fu et al. (2021)	HPFS (United States)	28 (max)	63.56 (mean)	25.23 (mean)	41,209 (41,209/0)	5,929	Cancer risk	Prostate cancer	Pathophysiologically confirmation	FFQ	Race, family history of prostate cancer, smoking status, total alcohol intake, PA, height, BMI, total energy intake, supplement use, vitamin E supplement use, aspirin use, PSA test in previous cycle, PSA testing in >50% of previous cycles.	7
Jin et al. (2021)	WHI (United States)	19.9 (median) 26 (max)	62.86 (mean)	27.46 (mean)	129,241 (0/129,241)	850	Cancer risk	Pancreatic cancer	Medical records	FFQ	Age, education, race/ethnicity, family history of T2D, smoking status, PA, BMI, total energy intake, supplement use, NSAID use, hormone use, comorbidity score, hormone therapy, dietary modification, and cholecystectomy status.	8
Sasamoto et al. (2021)	NHS, NHS-II (United States)	32 (max)	40.16 (median)	23.56 (median)	155,561 (0/155,561)	312	Cancer risk	Ovarian cancer	Medical records	FFQ	Parity, family history of breast or ovarian cancer, smoking status, total alcohol intake, BMI, aspirin use, oral contraceptive use, tubal ligation, hysterectomy, hormone therapy, hormone therapy use, and ovarian cancer risk factors a priority including menopausal status.	5
Yang et al. (2021)	HPFS, NHS (United States)	25.6 (mean) 28 (max)	63.85 (mean)	24.5 (mean)	119,316 (49,261/70,055)	142	Cancer risk	Liver cancer	Pathophysiologically confirmation	FFQ	Age, gender, race, smoking status, PA, total energy intake, and aspirin use.	6
Jin et al. (2023)	WHI (United States)	19.86 (median) 26 (max)	62.99 (mean)	27.56 (mean)	115,830 (0/115,830)	429	Cancer risk	kidney cancer	Pathophysiologically confirmation	FFQ	Education, diabetes status, family history of cancer, smoking status, total alcohol intake, PA, BMI, total energy intake, coffee/tea, supplement use, oral contraceptive duration, comorbidity score, hormone therapy, hormone use, and baseline lung disease.	8
Jin et al. (2023)	WHI (United States)	17.8 (median) 26 (max)	63.2 (mean)	27.59 (mean)	112,468 (0/112,468)	18,768 (total): 5,102 (colorectal) 4,393 (breast) 403 (endometrial) 260 (ovarian) 2,116 (lung)	Cancer risk	Total cancer and cite-specific cancer (colorectal, breast, endometrial, ovarian, and lung)	Pathophysiologically confirmation	FFQ	Age, race/ethnicity, education, family history of cancer, smoking status, total alcohol intake, PA, BMI, total energy intake, coffee/tea, supplement use, NSAID use, oral contraceptive use, comorbidity score, hormone therapy, baseline hormone use, baseline lung disease, and baseline cardiovascular disease.	7
Lee et al. (2023)	HPFS, NHS and CRC Nested (United States)		58.2 (mean)		7,888 (957/931)	3,199	Cancer risk	Colorectal cancer	Medical records	FFQ	Age, time of blood collection, race/ethnicity, family history of colorectal cancer, smoking status, total alcohol intake, PA, BMI, total energy intake, coffee intake, aspirin use, endoscopy status, and menopausal status.	6
Long et al. (2023)	NIH-AARP (United States)	15.5 (median) 16 (max)	61.55 (mean)	27.06 (mean)	485,931 (290,621/19,5310)	635	Cancer risk	Liver cancer	Medical records	FFQ	Age, gender, race, education, history of diabetes, smoking status, total alcohol intake, PA, BMI, total energy intake, and aspirin use.	8
Romanos-Nanclares et al. (2023)	NHS, NHS-II (United states)	NHS (32 max), NHS-II (26 max)	43.33 (mean)	25.27 (mean)	133,756 (0/133,756)	1,565	Cancer risk	Endometrial cancer	Pathophysiologically confirmation	FFQ	Age, parity, family history of endometrial cancer, smoking status, PA, BMI, total energy intake, oral contraceptive use, menopausal status, age at menarche, age at menopause, and postmenopausal hormone use.	8
Wang et al. (2023)	HPFS, NHS, NHS-II (United States)	24 (median) 30 (max)			218,181	3,428	Cancer risk	Colorectal cancer	Pathophysiologically confirmation	FFQ	Age, calendar time, family history of colorectal cancer, smoking status, total alcohol intake, PA, total energy intake, multivitamin use, aspirin use, NSAID use, postmenopausal hormone use for women, and history of colonoscopy or sigmoidoscopy.	5
Zhang et al. (2023)	WHI (United States)	22.1 (median) 28 (max)	63.4 (mean)	27.2 (mean)	78,356 (0/78,356)	176	Cancer risk	Liver cancer	Pathophysiologically confirmation	FFQ	Age, race/ethnicity, education, diabetes status, hypertension status, family history of cancer, smoking status, total alcohol intake, PA, BMI, total energy intake, NSAID use, hormone replacement therapy, liver disease, and the AHEI.	6
Liu et al. (2017)	HPFS, NHS (United States)	HPFS (26 max), NHS (28 max)	52.21 (mean)	25.25 (mean)	1,120	178	Mortality	Colorectal cancer	National Death Index	FFQ	Age, year of diagnosis, family history of colorectal cancer, smoking status, total alcohol intake, PA, BMI, aspirin use, EDIP scores, tumor stage, tumor location, and tumor differentiation.	9
Lee et al. (2020)	HPFS, NHS (United States)	HPFS (28 max), NHS (30 max)	70.81 (mean)	26.77 (mean)	423	295	Mortality	Multiple myeloma	National Death Index	FFQ	Age, year of diagnosis, time between FFQ return date and multiple myeloma diagnosis, BMI, total energy intake, and comorbidity score.	8
Fu et al. (2021)	HPFS (United States)	28 (max)	63.56 (mean)	25.23 (mean)	41,209 (41,209/0)	667	Mortality	Prostate cancer	National Death Index	FFQ	Race, family history of prostate cancer, smoking status, total alcohol intake, PA, height, BMI, total energy intake, supplement use, vitamin E supplement use, aspirin use, PSA test in previous cycle, and PSA testing in >50% of previous cycles.	7
Yuan et al. (2021)	HPFS, NHS (United States)	28 (max)	72.8 (mean)	26.1 (mean)	1,153 (480/673)	1,118	Mortality	Pancreatic cancer	National Death Index	FFQ	Age, diagnosis period, gender, race/ethnicity, diabetes status, smoking status, BMI, total energy intake, and cancer stage.	7
Longlais et al. (2022)	CaPSURE (United States)	20 (max)	64.43 (mean)	27.51 (mean)	2,447 (2,447/0)	73	Mortality	Prostate cancer	National Death Index	FFQ	Age, time between diagnosis and questionnaire completion date, race, family history of prostate cancer in brother or father, smoking status, total alcohol intake, PA, BMI, total energy intake, supplement use, CaPSURE clinical site, tumor stage at diagnosis, Gleason at diagnosis, PSA at diagnosis, and primary treatment.	6
Li et al. (2022)	US NHANES (United States)	16 (max)	47.3 (mean)	28.58 (mean)	40,074 (19,084/20990)	1,068	Mortality	Total cancer	National Death Index	24-h recalls	Gender, race/ethnicity, education, marital status, ratio of family income to poverty, diabetes status, smoking status, BMI and total energy intake.	8
Sasamoto et al. (2022)	NHS, NHS-II (United States)	40 (max)	62 (median)		783 (0/783)	402	Mortality	Ovarian cancer	National Death Index	FFQ	Age, year of diagnosis, smoking status, BMI, total energy intake, aspirin and non-aspirin, NSAID use, and histology stage.	7
Ugai et al. (2022)	HPFS, NHS (Unites States)	16.6 (median) 34 (max)	69.6 (mean)	25.9 (mean)	2,829 (1785/1044)	392	Mortality	Colorectal cancer	National Death Index	FFQ	Age, year of diagnosis, family history of colorectal cancer, smoking status, total alcohol intake, PA, BMI, total energy intake, aspirin use, pre-diagnosis EDIP scores, tumor location, and tumor differentiation.	7
Jin et al. (2023)	WHI (United States)	19.86 (median) 26 (max)	62.99 (mean)	27.56 (mean)	117,870 (0/117870)	113	Mortality	kidney cancer	National Death Index	FFQ	Education, family history of cancer, smoking status, total alcohol intake, PA, total energy intake, coffee/tea, supplement use, oral contraceptive duration, comorbidity score, baseline hormone therapy ever, hormone use, and baseline lung disease.	8

EDIP, Empirical Dietary Inflammatory Pattern; HPFS, Health Professionals Follow-up Study; NHS, Nurses’ Health Study; PLCO cancer cohort, Prostate Lung Colorectal Ovarian cancer cohort; WHI, Women’s Health Initiative; CRC Nested, colorectal cancer Nested; NIH-AARP, National Institutes of Health–American Association of Retired Persons Diet Health; CaPSURE, Cancer of the Prostate Strategic Urologic Research Endeavor; NHANES, National Health and Nutrition Examination Survey; FFQ, Food Frequency Questionnaire; PA, physical activity; BMI, body mass index; NSAIDs, non-steroidal anti-inflammatory drugs; PSA, prostate-specific antigen; T2D, type 2 diabetes; AHEI, Alternative Healthy Eating Index.

### Cancer incidence

Eighteen studies evaluated the EDIP–cancer risk relationship with seven categories of cancer types, including gastrointestinal cancers [colorectal (15, 30, 32, 33, 45, 47), liver (41, 46, 48), pancreatic (39)], urological cancers [prostate (36, 38), bladder cancer (34), kidney cancer (19)], ovarian cancer [(15, 31, 40), endometrial cancer (15, 16), breast cancer (15), multiple myeloma (35), and lung cancer (15)]. These studies enrolled 2,662,531 participants, ranging from 1,048 to 485,931. Over a 16- to 32-year follow-up period, a total of 37,091 cases of cancer were documented, including 13,196 cases of colorectal cancer, 953 cases of liver cancer, 850 cases of pancreatic cancer, 10,105 cases of prostate cancer, 1,042 cases of bladder cancer, 429 cases of kidney cancer, 478 cases of multiple myeloma, 4,393 cases of breast cancer, 1,968 cases of endometrial cancer, 1,561 cases of ovarian cancer, and 176 cases of lung cancer. These studies employed a food frequency questionnaire (FFQ) to calculate the EDIP score. The mean age and BMI at baseline ranged from 38.43 to 63.85 and 24.5 to 27.59, respectively. Cancer incidence was obtained from medical reports in nine studies (30, 31, 33–35, 39, 40, 45, 46), and nine studies from pathology reports (15, 16, 19, 32, 36, 38, 41, 47, 48). The majority of the included studies controlled for some conventional risk factors, including total energy intake (*n* = 17), supplement use (*n* = 9), alcohol consumption (*n* = 11), smoking (*n* = 15), physical activity (*n* = 13), and family history of cancer (*n* = 13).

### Cancer-specific mortality

Nine studies assessed the association between adherence to the EDIP and the risk of cancer-specific mortality (17–19, 30, 37, 38, 42–44), covering seven categories of cancer-specific mortality, including colorectal cancer (30, 44), prostate cancer (38, 43), pancreatic cancer (42), multiple myeloma (35), ovarian cancer (40), and kidney cancer (19). The studies included a total of 20,819 participants, ranging from 423 to 117,870. During a follow-up period spanning 16–40 years, a total of 5,006 cancer-specific mortality cases were recorded. These cases included 570 cases of colorectal cancer mortality, 740 cases of prostate cancer mortality, 1,118 cases of pancreatic cancer mortality, 295 cases of multiple myeloma mortality, 1,102 cases of ovarian cancer mortality, and 113 cases of kidney cancer mortality. Of the nine studies that evaluated the EDIP–cancer-specific mortality relationship, eight utilized the FFQ (18, 19, 30, 37, 38, 42–44), and one employed a 24-h recall questionnaire (17) to calculate the EDIP. The mean age and BMI of the participants at baseline exhibited a range between 47.3 and 72.3 years and 25.23 and 28.58 years, respectively. Cancer-specific mortality was obtained from the national death index in all included studies. Most of the included studies controlled for several conventional risk factors, including total energy intake (*n* = 8), supplement usage (*n* = 3), alcohol consumption (*n* = 5), smoking (*n* = 7), physical activity (*n* = 6), as well as family history of cancer (*n* = 4).

### EDIP and cancer incidence

**Figure 2:**
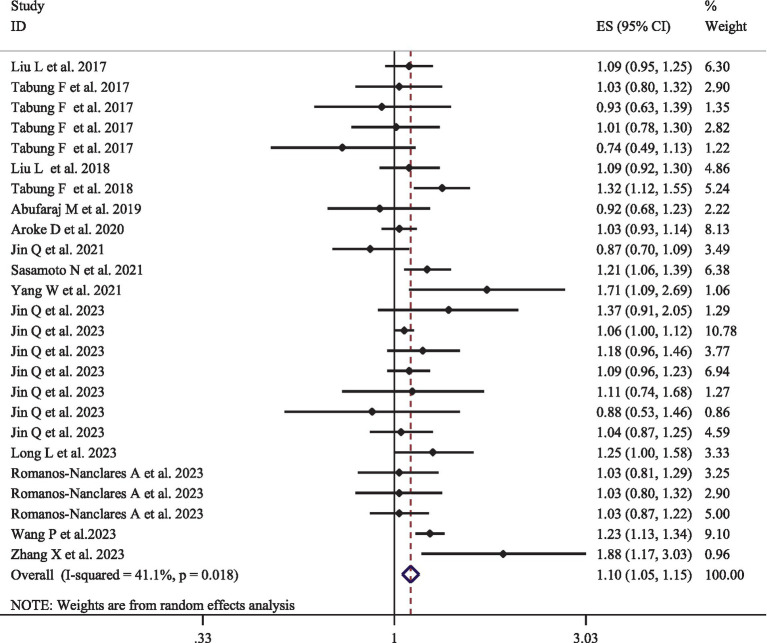
Forest plots with overall multi-variable adjusted effect sizes from the random-effects meta-analysis of the highest compared to lowest adherence to empirical dietary inflammatory pattern and the risk of cancer.

Figure 2 provides the pooled multivariate-adjusted ESs from the random-effects meta-analysis of the highest compared to the lowest categories of EDIP adherence and the risk of cancer. Twenty-five ES from 14 studies (15, 16, 30–34, 36, 39–41, 46–48) were included. The pooled ES was 1.10 (95% CI: 1.05–1.15), with a medium degree of heterogeneity (*I*^2^ = 41.1; *P*-heterogeneity = 0.018). Subgroup analysis showed that the quality of the studies and adjustment for alcohol consumption were all potential sources of heterogeneity (Table 3). The increased risk of cancer incidence also remained significant after stratification by age (<55, >55), gender (men/women), outcome assessment method (medical report/pathologic report), BMI (normal weight/overweight or obese), baseline T2DM status, quality of studies, and adjustment for total energy intake, supplement use, alcohol consumption, smoking status, physical activity, and family history of cancer (Table 3). In addition, according to the pooled stratified analysis by the number of participants, a significant association was found between greater adherence to the EDIP and an increased risk of cancer among studies with more than 100,000 participants. However, no significant association was observed for studies with less than 100,000 participants (Table 3). Follow-up duration stratified analysis also showed that a significant association was observed between greater EDIP adherence and an increased risk of cancer among studies with more than 20 years of follow-up. However, no significant association was observed for studies with less than 20 years of follow-up (Table 3).

**Table 3:** Subgroup analysis of the association between highest compared to lowest adherence to the empirical dietary inflammatory pattern and risk of cancer incidence (*n* = 2,662,531).

			Meta-analysis	Heterogeneity	
Subgroup		Effect sizes (*n*)	Effect size (95% CI)	*I*^2^ (%)	*P*-value for heterogeneity between subgroups
Age, years					
<55		8	1.08 (1.01–1.16)	0.3	0.97
>55		15	1.08 (1.04–1.12)	43.5	
Gender					
Men		13	1.03 (1.01–1.06)	84.9	0.84
Women		40	1.04 (1.02–1.05)	37.6	
No. of participants					
<100,000		7	1.03 (0.92–1.15)	44	0.21
>100,000		18	1.10 (1.07–1.14)	32	
Follow-up duration, years					
<20		4	1.04 (0.95–1.14)	44.2	0.14
>20		21	1.11 (1.07–1.14)	40.6	
Outcome assessment method					
Medical report		10	1.11 (1.04–1.18)	51.3	0.78
Pathology report		15	1.10 (1.06–1.14)	36.9	
Body mass index					
Normal weight		20	1.04 (1.01–1.07)	39.6	0.97
Overweight or obese		29	1.04 (1.02–1.06)	58.6	
Baseline T2DM status					
No		10	1.03 (1.01–1.04)	58.8	0.20
Yes		12	1.07 (1.00–1.14)	14.2	
Quality of studies					
Low quality		5	1.26 (1.18–1.34)	26.4	<0.01
High quality		20	1.05 (1.02–1.09)	0	
Adjustment for major confounders					
Total energy intake	No	1	1.21 (1.06–1.39)	–	0.15
	Yes	24	1.09 (1.06–1.13)	40.5	
Supplement use	No	11	1.10 (1.06–1.15)	38.2	0.59
	Yes	14	1.08 (1.02–1.15)	48.5	
Alcohol consumption	No	11	1.01 (0.95–1.08)	0	0.01
	Yes	14	1.13 (1.09–1.17)	40.5	
Smoking status	No	5	1.03 (0.93–1.14)	0	0.20
	Yes	20	1.11 (1.07–1.14)	46.9	
Physical activity	No	6	1.07 (0.98–1.18)	36.8	0.63
	Yes	19	1.10 (1.07–1.14)	44.8	
Family history of cancer	No	3	1.18 (1.00–1.40)	63.9	0.38
	Yes	22	1.10 (1.06–1.13)	39	

According to the sensitivity analysis using the random-effects model, the overall ESs regarding the association between greater adherence to the EDIP and the risk of cancer did not depend on a single study (95% CI: 1.04–1.16) (Supplementary Figure S1).

The meta-regression association between greater adherence to the EDIP and the risk of cancer based on age and BMI is presented in Supplementary Figures S2, S3. According to these findings, age and BMI were not significant sources of heterogeneity in these associations (all *p*-values >0.05).

Supplementary Figure S4 provides an assessment of publication bias, displaying the funnel plots of ESs for greater adherence to the EDIP and the risk of cancer, which shows no asymmetry. It also includes results from Egger’s and Begg’s tests. When the funnel plot was visually inspected, no evidence of publication bias was observed, which was also confirmed using Egger’s and Begg’s tests (all *p*-values >0.05).

Supplementary Figure S5 presents the pooled multivariate-adjusted ESs from the random-effects meta-analysis of the per-SD increases in adherence to the EDIP and the risk of cancer. Fifteen ESs from seven studies (15, 16, 35, 36, 38, 39, 45) were included. The pooled ES was 1.03 (95% CI: 1.01–1.06), with a high degree of heterogeneity (*I*^2^ = 62.3; *P*-heterogeneity <0.01).

The pooled multivariable-adjusted ES from the random-effects meta-analysis of the association between the highest compared to the lowest adherence to the EDIP and the risk of site-specific cancer are represented in Table 4. There was a significant association between greater adherence to the EDIP and the risk of colorectal cancer (ES: 1.19; 95% CI: 1.11–1.15; *I*^2^ = 41.1) and liver cancer (ES: 1.48; 95% CI: 1.14–1.94; *I*^2^ = 36.9). However, no significant association between greater adherence to the EDIP and the risk of ovarian cancer (ES: 1.03; 95% CI: 0.90–1.19; *I*^2^ = 32.6) and endometrial cancer (ES: 1.04; 95% CI: 0.87–1.25; *I*^2^ = 0) was found (Table 4).

**Table 4:** Empirical dietary inflammatory pattern in relation to site-specific cancer incidence based on analysis of the highest compared to lowest adherence.

Cancer type (number of cases/total sample)	Effect sizes (*n*)	Effect size (95% CI)	*I*^2^ (%)	*P*-heterogeneity
All cancers (37,091/2,662,531)	25	1.10 (1.05–1.15)	41.1	0.018
Site-specific cancer				
Colorectal cancer (9,997/700,565)	5	1.19 (1.11–1.27)	15	0.32
Ovarian cancer (1,561/640,657)	6	1.03 (0.90–1.19)	32.6	0.19
Bladder cancer (1,042/342,264)	1	0.92 (0.68–1.24)	–	–
Prostate cancer (10,105/49,317)	1	1.03 (0.93–1.14)	–	–
Pancreatic cancer (129,241)	1	0.87 (0.70–1.09)	–	–
Liver cancer (953/683,603)	3	1.48 (1.14–1.94)	36.9	0.20
Kidney cancer (429/115,830)	1	1.37 (0.91–2.06)	–	–
Breast cancer (4,393/112,468)	1	1.09 (0.96–1.23)	–	–
Endometrial cancer (1968/246,224)	4	1.04 (0.92–1.16)	0	0.90
Lung cancer (2,116/112,468)	1	1.04 (0.87–1.25)	–	–

### EDIP and cancer-specific mortality

**Figure 3:**
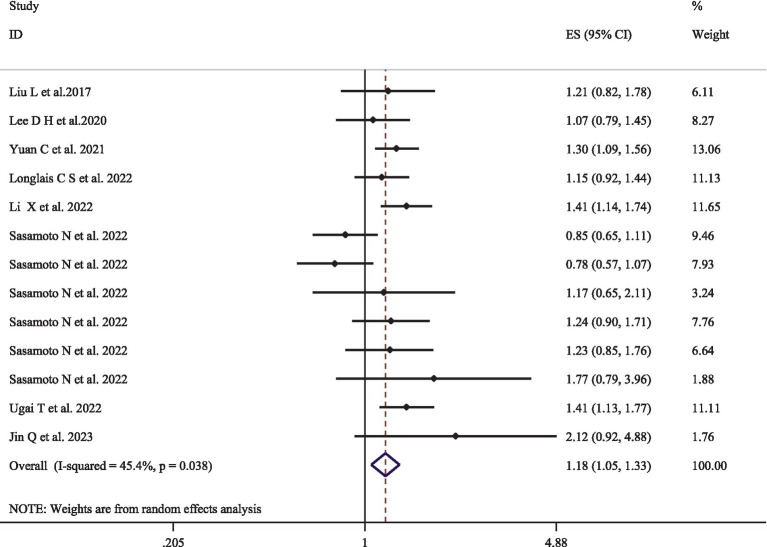
Forest plots with overall multi-variable adjusted effect sizes from the random-effects meta-analysis of the highest compared to lowest adherence to empirical dietary inflammatory pattern and the risk of cancer-specific mortality.

Figure 3 represents the pooled multivariate-adjusted ES from the random-effects meta-analysis of adherence to the highest compared to the lowest EDIP and the risk of cancer-specific mortality. Thirteen ESs from the eight studies (17–19, 30, 37, 42–44) were included. The pooled ES was 1.18 (95% CI: 1.05–1.33), with a medium degree of heterogeneity (*I*^2^ = 45.4; *P*-heterogeneity = 0.038). Subgroup analysis showed that the number of participants and adjustment for physical activity and family history of cancer were all potential sources of heterogeneity (Supplementary Table S3). The increased risk of cancer-specific mortality also remained significant after stratification by age (<55, >55), gender (men/women), number of participants (<100,000, >100,000), follow-up duration (<20 years, >20 years), dietary intake assessment method (FFQ, 24-h recall questionnaire), and adjustment for total energy intake, alcohol consumption, smoking status, physical activity, and family history of cancer (Supplementary Table S3).

Based on sensitivity analysis, the overall ES regarding the association between greater adherence to the EDIP and the risk of cancer-specific mortality did not rely on a single study (95% CI: 1.04–1.16) (Supplementary Figure S6).

The meta-regression association between greater adherence to the EDIP and the risk of cancer-specific mortality based on age and BMI is provided in Supplementary Figures S7, S8. The results of the analysis revealed that neither age nor BMI were found to be significant sources of heterogeneity (all *p*-values >0.05).

Supplementary Figure S9 presents a thorough evaluation of publication bias by displaying the funnel plots of ESs comparing greater adherence to the EDIP and the risk of cancer-specific mortality without any asymmetry, along with Egger’s and Begg’s tests. Upon visual inspection of the funnel plot, no evidence of publication bias was found, which was further confirmed using Egger’s and Begg’s tests (all *p*-values >0.05).

Supplementary Figure S10 shows the multivariate-adjusted ES from the random-effects meta-analysis of adherence to per-SD increases in EDIP adherence and the risk of cancer-specific mortality. Five ESs from the five studies (17, 19, 35, 38, 43) were included in the meta-analysis of the per-SD increases in adherence to EDIP and the risk of cancer-specific mortality. The pooled ES was 1.14 (95% CI: 1.02–1.28), with a high degree of heterogeneity (*I*^2^ = 73.4; *P*-heterogeneity <0.01).

## Discussion

This systematic review and meta-analysis aimed to assess the association between greater adherence to the EDIP and the risk of cancer and cancer-specific mortality. Our results showed that greater adherence to the EDIP was significantly associated with a 10% increase in risk of cancer incidence with a medium degree of heterogeneity. Our subgroup analysis showed that the significant positive association between greater adherence to the EDIP and the risk of cancer remained unchanged except for studies with fewer than 100,000 participants and less than 20 years of follow-up. Our findings also indicate that higher adherence to the EDIP is significantly associated with an increased risk of colorectal and liver cancer. However, we did not find a significant association between EDIP adherence and the risk of ovarian or endometrial cancer. We showed that greater adherence to the EDIP was significantly associated with an 18% increase in the risk of cancer-specific mortality. Furthermore, we found that the significant positive association between greater adherence to the EDIP and risk of cancer mortality remained unchanged except for studies adjusted for supplement intake.

The current study’s findings of an association between dietary inflammatory potential and the risk of cancer are consistent with previous studies. In 2018, a meta-analysis that pooled data from 44 epidemiologic studies involving more than one million participants found that the highest adherence to the DII was significantly associated with a 58% increased risk of total cancer compared to the lowest adherence (49). Additionally, the aforementioned meta-analysis demonstrated that for each SD increase in the DII, the risk of cancer significantly increased by 13% (49). Another meta-analysis which pooled the 38 observational studies showed that a higher level of DII adherence was significantly associated with a 32% higher risk of total cancer incidence (50). According to our findings, we showed that greater adherence to the EDIP was significantly associated with a higher risk of colorectal cancer and liver cancer by 19 and 48%, respectively. In this context, a recent meta-analysis that pooled data from 12 studies demonstrated that greater adherence to the DII was significantly associated with a 16% higher risk of colorectal cancer (51). Recently, another meta-analysis evaluating the association between adherence to pro-inflammatory diets and the risk of liver cancer showed that greater adherence to pro-inflammatory diets was significantly associated with a 2.35-fold increased risk of liver cancer (52). Our study did not reveal a significant association between adherence to the EDIP and the risk of ovarian or endometrial cancer. In this context, in 2021, a meta-analysis conducted by Yang et al. evaluated adherence to pro-inflammatory diets (including four studies considered the DII as the exposure and two studies considered the EDIP as the exposure) and the risk of ovarian cancer (53). In line with our findings, according to the aforementioned meta-analysis, no significant association between greater adherence to the EDIP and the risk of ovarian cancer was found. Furthermore, a recent meta-analysis evaluated adherence to the DII and the risk of gynecological cancers (54). In contrast to our findings, this meta-analysis showed that greater adherence to the DII was significantly associated with a higher risk of endometrial cancer. It is important to note that this meta-analysis pooled data from two studies to evaluate this association, which may lead to limited statistical power, and future studies are needed to investigate associations between adherence to pro-inflammatory diets and the risk of endometrial cancer.

Findings of an association between adherence to the EDIP and the risk of cancer-specific mortality from the current study are consistent with previous studies. In 2022, a meta-analysis evaluated the association between adherence to the DII and the risk of all-cause and cause-specific mortality demonstrated that the highest adherence to the DII compared to the lowest one was significantly associated with a 7% higher risk of cancer-specific mortality (12). In addition, the authors showed that per 1-unit DII increase was significantly associated with a 2% higher risk of cancer-specific mortality (12). Furthermore, Zahedi et al., in the framework of the meta-analysis, showed that higher adherence to the DII was significantly associated with a 16% higher risk of cancer-specific mortality (50).

Several potential mechanisms may explain the association between inflammation and an increased risk of cancer as well as cancer-specific mortality. It has been shown that there is a strong association between chronic inflammation and different types of cancers (55–57). Research findings suggest that approximately 20% of all cancer-related deaths can be attributed to inflammation and chronic infections (58). However, the mechanisms underlying these relationships have not been fully characterized. The infiltration of leukocytes, accumulation of macrophages, and activation of transcription factors, particularly nuclear factor kappa-light-chain enhancer of activated B cells (NF-κB), are pathways that can bring about inappropriate gene expression, enhanced cell proliferation, and resistance to apoptosis in initiated cells (58). These mechanisms may ultimately lead to the development and dissemination of tumor cells (58). In this context, it is vital to recognize that the activation of NF-κB pathways serves as a crucial mechanism, playing a pivotal role in mediating the link between inflammation and carcinogenesis (59).

The possible mechanisms through which dietary features are associated with inflammatory status remain incompletely specified. In the EDIP scoring system, processed meat, red meat, organ meat, fish, refined grains, high-energy beverages, and tomato intake are pro-inflammatory components (14). Red meat and processed meat, being rich sources of saturated fat and heme iron, can be significant dietary risk factors contributing to increased risk of cancer and cancer-specific mortality (60–62). Red and processed meats, characterized by higher levels of saturated and trans fatty acids, have been linked to heightened oxidative stress and elevated plasma concentrations of inflammatory biomarkers, notably C-reactive protein (63, 64). Additionally, saturated fatty acids activate inflammatory genes through mechanisms such as NF-κB activation, protein kinase C, and mitogen-activated protein kinases (65). Moreover, trans fatty acids increase the activation of the TNF system, leading to heightened inflammation (66). The heme iron present in red and processed meat has been shown to have detrimental effects on our bodies (60, 62, 67). It can lead to cell damage, cell death, increased growth of epithelial cells, oxidative damage to lipids, generation of free radicals, formation of DNA adducts in epithelial cells, and promotion of the formation of N-nitroso compounds, which are known to contribute to the development of cancer (67). Furthermore, the consumption of refined grains and high-energy beverages, due to their higher energy and carbohydrate content, may result in weight gain and exacerbate inflammatory conditions (68–70). According to the EDIP components, fish (other than dark meat fish) and tomatoes have a positive association with inflammatory markers, whereas pizza was inversely related (14). This may suggest that it pertains to fish preparation methods, although this information was not gathered by Tabung et al. (14). For example, fish that is well-done, browned, fried, grilled, or barbecued may be more pro-inflammatory and associated with a higher risk of chronic diseases (71). The oils commonly used for deep frying are low in n-3 fatty acids due to the oxidation of these acids (72). Additionally, prior to the regulation of trans fats in the United States, these oils also contained high amounts of trans fats, which are known to be pro-inflammatory (72). Three clinical trials were conducted to investigate the effect of tomato intake on the serum level of systemic inflammatory markers, yielding conflicting findings (73–75). One study reported that tomato juice supplementation can significantly increase the serum levels of adiponectin and reduce inflammatory adipokine levels independently of body fat reduction (74). However, it is worth noting that several other studies have not demonstrated a significant impact of tomato consumption on reducing the serum levels of IL-6, CRP, and other inflammatory markers (73, 75). It is possible that the potential benefits of a tomato-rich diet may not be directly related to the inflammation process. It should be noted that tomato paste has 2.5–4 times greater bioavailable lycopene content compared to fresh tomatoes, and lycopene has shown anti-inflammatory properties (53). Lycopene has demonstrated anti-inflammatory properties, which could clarify the reverse connection between pizza and inflammatory markers (76).

We acknowledge that our study has its strengths and limitations. This is the first systematic review and meta-analysis evaluating the association between adherence to EDIP and the risk of cancer and cancer-specific mortality using data from 24 prospective cohort studies. We performed subgroup analysis based on various confounding factors to elucidate the sources of variation among the included studies. In addition, most of the included studies controlled for factors that could affect the association between adherence to the EDIP and the risk of cancer and cancer-specific mortality. However, there are certain limitations that need to be mentioned. First, the self-reported nature of dietary intake using the FFQ in the included studies, coupled with the lack of repeated measurements of dietary intake in the majority of the studies, may lead to measurement errors and misclassifications; second, even after adjusting for various confounding factors among the included studies, it is not possible to entirely rule out unmeasured or residual confounding factors; third, due to a limited number of included studies in some subgroup analyses based on outcome site-specific cancer, we were unable to perform a meta-analysis for all site-specific cancers; and finally, it should be noted that the EDIP score was developed and validated using data from the Nurses’ Health Study in the United States, which restricts its application to other countries with differing medical and dietary characteristics.

## Conclusion

In conclusion, the present systematic review and meta-analysis suggest that a diet with higher inflammatory properties, represented by the EDIP, is associated with an increased risk of cancer and cancer-specific mortality. It may be beneficial to validate the EDIP in other countries and assess the relationship between adherence to the EDIP and the risk of all site-specific cancers.

## Data Availability

The original contributions presented in the study are included in the article/Supplementary material, further inquiries can be directed to the corresponding author.
