# Supplementary material for: The association between empirical dietary inflammatory pattern and risk of cancer and cancer-specific mortality: a systematic review and meta-analysis of prospective cohort studies
Source: Front Nutr. 2024 Oct 18;11:1462931. doi: 10.3389/fnut.2024.1462931 (PMC11527705; doi:10.3389/fnut.2024.1462931)
Supplement: Supplementary file 1 [file Table_1.docx]

**Online Supplementary Material**

The association between Empirical dietary inflammatory index and risk of cancer and cancer-specific mortality: a systematic review and meta-analysis of prospective studies

**Supplementary file description:**

Search strategy used in this study is shown in supplementary table 1 which describes the key terms used to search in each database. The quality assessment of included studies is provided in Supplementary Table 2. Subgroup analysis of the association between highest compared to lowest adherence to the empirical dietary inflammatory pattern and risk of cancer mortality is provided in supplementary Table 3. Sensitivity analysis is employed to evaluate the robustness of the findings (Supplementary Figures 1 and 6). Meta-regression is applied to find the potential source of heterogeneity based on age and BMI of participants (Supplementary Figures 2, 3, 7, and 8). Funnel plot is used to assess the potential role of publication bias based on visual or analytical detection of asymmetry (Supplementary Figures 4 and 9). The pooled ESs from the random-effect meta-analysis of adherence to per-SD increases in EDIP adherence and risk of cancer and cancer-specific mortality are provided in supplementary figures 5 and 10.

**Supplementary Table 1.** The structure of key terms used to search international databases (Search date: 03/27/2024)

| PubMed | Scopus | Web of Science |
| --- | --- | --- |
| (“Empirical dietary index”[tiab] OR "Empirical Dietary Inflammatory Index"[tiab] OR EDII[tiab] OR "empirical dietary inflammatory pattern"[tiab] OR EDIP[tiab]) AND ("Neoplasms"[Mesh] OR "Carcinogens"[Mesh] OR "Leukoplakia"[Mesh] OR "Hyperplasia"[Mesh] OR cancer*[tiab] OR neoplasm*[tiab] OR carcinoma*[tiab] OR tumor*[tiab] OR carcinogen*[tiab] OR tumour*[tiab] OR adenoma*[tiab] OR mortality*[tiab] OR death*[tiab] OR survival*[tiab]) AND (Cohort*[tiab] OR case-cohort[tiab] OR "nested case-control"[tiab] OR population-based[tiab] OR incident*[tiab] OR incidence*[tiab] OR Prospective[tiab] OR Longitudinal[tiab] OR Observational[tiab] OR Follow-Up[tiab] OR “Odds ratio”[tiab] OR "Relative risk"[tiab] OR "Hazard ratio"[tiab] OR cox[tiab]) AND 2016/01/01:2024/02/29[Date - Publication] | TITLE-ABS-KEY(“Empirical dietary index” OR "Empirical Dietary Inflammatory Index" OR EDII OR "empirical dietary inflammatory pattern" OR EDIP) AND TITLE-ABS-KEY(neoplasm* OR cancer* OR tumor* OR carcinoma* OR carcinogen* OR leukoplakia* OR dysplasia* OR hyperplasia* OR malignancy* OR adenoma* OR mortality* OR death* OR survival*) AND TITLE-ABS-KEY(Cohort* OR case-cohort OR "nested case-control" OR population-based OR incident* OR incidence* OR Prospective OR Longitudinal OR Observational OR Follow-Up OR “Odds ratio” OR "Relative risk" OR "Hazard ratio" OR cox) AND ( ( PUBYEAR > 2015 AND PUBYEAR < 2024 ) OR PUBDATETXT ( "January 2024" ) OR PUBDATETXT ( "February 2024" )) | TS=(“Empirical dietary index” OR "Empirical Dietary Inflammatory Index" OR EDII OR "empirical dietary inflammatory pattern" OR EDIP) AND TS=( neoplasm* OR cancer* OR tumor* OR carcinoma* OR carcinogen* OR leukoplakia* OR dysplasia* OR hyperplasia* OR malignancy* OR adenoma* OR mortality* OR death* OR survival*) AND TS=(Cohort* OR case-cohort OR "nested case-control" OR population-based OR incident* OR incidence* OR Prospective OR Longitudinal OR Observational OR Follow-Up OR “Odds ratio” OR "Relative risk" OR "Hazard ratio" OR cox) AND PY=(2016-2024) |

| **Supplementary Table 2.** The quality assessment of studies included in this study on the EDIP and risk of cancer and cancer-specific mortality.^1^ | | | | |
| --- | --- | --- | --- | --- |
| Study | Selection | Comparability | Outcome | NOS Score |
| **Cancer Risk** |  |  |  |  |
| Liu L et al. (2017) | **** | ** | *** | 9 |
| Tabung F et al. (2017) | **** | ** | *** | 9 |
| Liu L et al. (2018) | *** | ** | *** | 8 |
| Tabung F et al. (2018) | *** | * | ** | 6 |
| Lee D H et al. (2019) | ** | ** | *** | 7 |
| Abufaraj M et al. (2019) | **** | * | ** | 7 |
| Aroke D et al. (2020) | *** | ** | ** | 7 |
| Fu B C et al. (2021) | *** | ** | ** | 7 |
| Jin Q et al. (2021) | **** | ** | ** | 8 |
| Sasamoto N et al. (2021) | ** | * | ** | 5 |
| Yang W et al. (2021) | *** | * | ** | 6 |
| Jin Q et al. (2023) | **** | ** | ** | 8 |
| Jin Q et al. (2023) | *** | ** | ** | 7 |
| Lee D et al. )2023( | ** | ** | ** | 6 |
| Long L et al. (2023) | **** | ** | ** | 8 |
| Romanos-Nanclares A et al. (2023) | **** | ** | ** | 8 |
| Wang P et al. (2023) | ** | * | ** | 5 |
| Zhang X et al. (2023) | ** | ** | ** | 6 |
| **Cancer-specific mortality risk** |  |  |  |  |
| Liu L et al. (2017) | **** | ** | *** | 9 |
| Lee D H et al.2020 | **** | ** | ** | 8 |
| Fu B C et al. (2021) | *** | ** | ** | 7 |
| Yuan C et al. (2021) | *** | ** | ** | 7 |
| Longlais C S et al. (2022) | *** | ** | * | 6 |
| Li X et al. (2022) | **** | ** | ** | 8 |
| Sasamoto N et al. (2022) | *** | ** | ** | 7 |
| Ugai T et al. (2022) | *** | ** | ** | 7 |
| Jin Q et al. (2023) | **** | ** | ** | 8 |
| ^1^ According to the Newcastle-Ottawa Scale (NOS) criteria (Ref. 22)  ^*^ One point | | | | |

| **Supplementary Table 3.** Subgroup analysis of the association between highest compared to lowest adherence to the empirical dietary inflammatory pattern and risk of cancer mortality. | | | | | |
| --- | --- | --- | --- | --- | --- |
| Characteristics | | Study estimates, no | Effect size (95%CI) | I^2^(%) | P-value for heterogeneity between subgroups |
| **Age, year** | |  |  |  |  |
| <55 | | 2 | 1.36 (1.13-1.64) | 0 | 0.14 |
| >55 | | 11 | 1.17 (1.07-1.27) | 48.3 |  |
| **Gender** | |  |  |  |  |
| Men | | 5 | 1.05 (1.01-1.10) | 0 | 0.56 |
| Women | | 10 | 1.07 (1.02-1.13) | 68.9 |  |
| **No. of participants** | |  |  |  |  |
| <100,000 | | 11 | 1.16 (1.06-1.27) | 42.5 | 0.05 |
| >100,000 | | 2 | 1.45 (1.18-1.77) | 0 |  |
| **Follow up duration, year** | |  |  |  |  |
| <20 | | 2 | 1.28 (1.10-1.49) | 40.5 | 0.33 |
| >20 | | 11 | 1.17 (1.07-1.29) | 48.3 |  |
| **Dietary intake method** | |  |  |  |  |
| Food frequency questionnaire | | 12 | 1.17 (1.07-1.27) | 43.2 | 0.10 |
| 24-recall | | 1 | 1.41 (1.14-1.74) | - |  |
| **Adjustment for major confounders** | |  |  |  |  |
| Total energy intake | No | 1 | 1.21 (0.82-1.78) | - | 0.96 |
|  | Yes | 12 | 1.20 (1.11-1.30) | 50 |  |
| supplement used | No | 11 | 1.20 (1.10-1.31) | 50.2 | 0.99 |
|  | Yes | 2 | 1.20 (0.97-1.49) | 48.1 |  |
| alcohol consumption | No | 9 | 1.16 (1.06-1.28) | 54.8 | 0.27 |
|  | Yes | 4 | 1.28 (1.11-1.48) | 2.5 |  |
| smoking status | No | 1 | 1.07 (0.79-1.45) | - | 0.44 |
|  | Yes | 12 | 1.21 (1.11-1.31) | 48.6 |  |
| physical activity | No | 8 | 1.11 (1.00-1.23) | 49.2 | 0.03 |
|  | Yes | 5 | 1.32 (1.17-1.49) | 0 |  |
| family history of cancer | No | 9 | 1.16 (1.06-1.28) | 54.8 | 0.04 |
|  | Yes | 4 | 1.28 (1.11-1.48) | 2.5 |  |

**Supplementary Figure 1.** Sensitivity analysis using the random effect model on the association between highest compared to lowest adherence to EDIP and risk of cancer incidence.

**Supplementary Figure 2.** Meta-regression plots of the association between highest compared to lowest adherence to empirical dietary inflammatory pattern and risk of cancer incidence based on the age of participants.

**Supplementary Figure 3.** Meta-regression plots of the association between highest compared to lowest adherence to empirical dietary inflammatory pattern and risk of cancer incidence based on the body mass index of participants.

**Supplementary Figure 4.** Funnel plot to assess publication bias on the association between highest compared to lowest adherence to empirical dietary inflammatory pattern and risk of cancer incidence.

**Supplementary Figure 5.** Forest plots with overall multi-variable adjusted effect sizes from the random-effect meta-analysis of the per-SD increases in the adherence to empirical dietary inflammatory pattern and risk of cancer incidence.

**Supplementary Figure 6.** Sensitivity analysis using the random effect model on the association between highest compared to lowest adherence to EDIP and risk of cancer specific mortality.

**Supplementary Figure 7.** Meta-regression plots of the association between highest compared to lowest adherence to empirical dietary inflammatory pattern and risk of cancer specific mortality based on the age of participants.

**Supplementary Figure 8.** Meta-regression plots of the association between highest compared to lowest adherence to empirical dietary inflammatory pattern and risk of cancer specific mortality based on the body mass index of participants.

**Supplementary Figure 9.** Funnel plot to assess publication bias on the association between highest compared to lowest adherence to empirical dietary inflammatory pattern and risk of cancer specific mortality.

**Supplementary Figure 10.** Forest plots with overall multi-variable adjusted effect sizes from the random-effect meta-analysis of the per-SD increases in the adherence to empirical dietary inflammatory pattern and risk of cancer specific mortality.
